# Prevalence and Risk of Dental Erosion in Patients with Gastroesophageal Reflux Disease: A Meta-Analysis

**DOI:** 10.3390/dj10070126

**Published:** 2022-07-05

**Authors:** Oleg O. Yanushevich, Igor V. Maev, Natella I. Krikheli, Dmitrii N. Andreev, Svetlana V. Lyamina, Filipp S. Sokolov, Marina N. Bychkova, Petr A. Beliy, Kira Y. Zaslavskaya

**Affiliations:** Moscow State University of Medicine and Dentistry Named after A.I. Evdokimov, 127473 Moscow, Russia; mail@msmsu.ru (O.O.Y.); igormaev@rambler.ru (I.V.M.); nataly0088@mail.ru (N.I.K.); svlvs@mail.ru (S.V.L.); phlppsokolov@gmail.com (F.S.S.); estetstom.fpdo@gmail.com (M.N.B.); p.bely@ncpharm.ru (P.A.B.); kiryonok@yandex.ru (K.Y.Z.)

**Keywords:** gastroesophageal reflux disease, reflux, dental erosion, acid erosion, erosive tooth wear

## Abstract

Aim: The present paper aims to systematize data concerning the prevalence and risk of dental erosion (DE) in adult patients with gastroesophageal reflux disease (GERD) compared to controls. Materials and methods: Core electronic databases, i.e., MEDLINE/PubMed, EMBASE, Cochrane, Google Scholar, and the Russian Science Citation Index (RSCI), were searched for studies assessing the prevalence and risk of DE in adult GERD patients with publication dates ranging from 1 January 1985 to 20 January 2022. Publications with detailed descriptive statistics (the total sample size of patients with GERD, the total sample size of controls (if available), the number of patients with DE in the sample of GERD patients, the number of patients with DE in the controls (if available)) were selected for the final analysis. Results: The final analysis included 28 studies involving 4379 people (2309 GERD patients and 2070 control subjects). The pooled prevalence of DE was 51.524% (95 CI: 39.742–63.221) in GERD patients and 21.351% (95 CI: 9.234–36.807) in controls. An association was found between the presence of DE and GERD using the random-effects model (OR 5.000, 95% CI: 2.995–8.345; I^2^ = 79.78%) compared with controls. When analyzing studies that only used validated instrumental methods for diagnosing GERD, alongside validated DE criteria (studies that did not specify the methodologies used were excluded), a significant association between the presence of DE and GERD was revealed (OR 5.586, 95% CI: 2.311–13.503; I^2^ = 85.14%). Conclusion: The meta-analysis demonstrated that DE is quite often associated with GERD and is observed in about half of patients with this extremely common disease of the upper gastrointestinal tract.

## 1. Introduction

Gastroesophageal reflux disease (GERD) is one of the most common gastrointestinal disorders, which is caused by a dysfunction of the motor-evacuation function of the gastroesophageal zone leading to spontaneous and regularly repeated retrograde reflux of the gastric and duodenal liquids into the esophagus [1,2]. According to a recent meta-analysis by Nirwan JS et al. in 2020—which summarized the results of 102 studies—the global prevalence of GERD is 13.98% (95% CI: 12.47–15.56) [3].

A characteristic feature of GERD is a chronic, recurrent pattern of symptoms that has a significant negative impact on the patient’s quality of life [2,4]. The classic clinical manifestations of the disease are heartburn, belching, and regurgitation; however, in some cases, GERD may be characterized by complex atypical symptoms, also referred to as extraesophageal syndromes [5,6]. In the largest prospective multicenter cohort study, i.e., ProGERD (*n* = 6215), atypical symptoms were detected in 32.8% of patients with heartburn [7]. According to the global Montreal Consensus (2006), a cough, laryngitis, bronchial asthma, and erosion of dental hard tissues of reflux etiology are extraesophageal syndromes that are significantly associated with GERD [8].

Dental erosion (DE) refers to non-carious lesions of the hard tissues of the tooth (mainly enamel and, in some cases, dentin) that are induced by a chemical reaction involving acids and that lead to demineralization processes independently of a bacterial factor [9,10]. DE leads to aesthetic defects and, in the case of prolonged progression, dentin exposure and the development of hypersensitivity, which has a negative impact on the quality of life [11,12]. According to the latest review, the average global incidence of DE among the adult population is 20–45% [13]. Moreover, on the epidemiological level, there has been an increase in the frequency of DE in all age groups, which may indicate an increasing influence of risk factors for this pathology in the population [14,15]. The genesis of DE is multifactorial and may be related to external acidifying factors (diet and lifestyle) and internal factors (chronic reflux of gastric contents into the oral cavity; recurrent vomiting) (Table 1) [10,13]. GERD is the most common trigger of DE, which is a result of the retrograde reflux of acidic gastric contents into the oral cavity [5,6,16,17,18]. According to several early systematic reviews, the incidence of DE in adult GERD patients is 32.5–38.96% [19,20]. Furthermore, various studies have noted that the higher the severity of erosive damage to the hard tissues of the teeth in GERD patients compared to controls [6,21]. To date, a large number of published studies on the prevalence of DE in patients with GERD have accumulated around the world, requiring systematization to objectify the global prevalence. The present paper aims to systematize data concerning the prevalence and risk of dental erosion (DE) in adult patients with gastroesophageal reflux disease (GERD) compared to controls.

## 2. Materials and Methods

### 2.1. Study Sources and Search

A search was carried out in MEDLINE/PubMed, EMBASE, Cochrane, Google Scholar, and the Russian Science Citation Index (RSCI) for studies published between 1 January 1985 and 20 January 2022 (inclusive) based on the analysis of titles and abstracts of entries within these databases. The following keyword combinations were used to search the MEDLINE/PubMed database: “dental erosion [Title/Abstract] OR dental erosions [Title/Abstract] OR acid erosions [Title/Abstract] OR erosive toothwear [Title/Abstract] AND reflux [Title/Abstract]”. The corresponding terms in English were used for searching in the Google Scholar and RSCI database.

### 2.2. Study Selection

The criteria for the meta-analysis were as follows: relevant publications in peer-reviewed periodicals in English or Russian; publications with detailed descriptive statistics, which allowed the resulting data to be included in a meta-analysis; studies in the adult population of patients with GERD. Studies conducted on specific patient populations (diseases and conditions that may affect the objectivity and comparability of data) were excluded from the analysis. In cases of duplicated results in two publications (from different or the same electronic database), one was selected for the final analysis. The methodological quality of each of the included studies was assessed using the Newcastle–Ottawa Scale (NOS).

### 2.3. Data Extraction

Two investigators (D.N.A. and F.S.S.) independently extracted data using standardized forms. The year of publication, country, methodology for diagnosing GERD, criteria for diagnosing DE, the total sample size of patients with GERD, the total sample size of controls (if available), the number of patients with DE in the sample of patients with GERD, and the number of patients with DE in the sample of controls (if available) were analyzed. Any disagreements were resolved by discussion until reaching a consensus.

### 2.4. Statistical Analysis

Statistical data processing was carried out using the specialized software MedCalc 20.023 (MedCalc Software, Ostend, Belgium) in Microsoft Windows 11 (Microsoft, Redmond, WA, USA). The results are presented as the pooled frequency of DE in GERD patients/controls and a 95% confidence interval (95% CI). Heterogeneity between different studies was assessed using Cochrane’s Q test and I^2^ test. Significant heterogeneity was noted for results at *p* < 0.05 and I^2^ > 50. The probability of a publication error was estimated by constructing a funnel plot and calculations according to the Begg–Mazumdar correlation test and Egger’s test.

## 3. Results

### 3.1. Search Results

**Figure 1:**
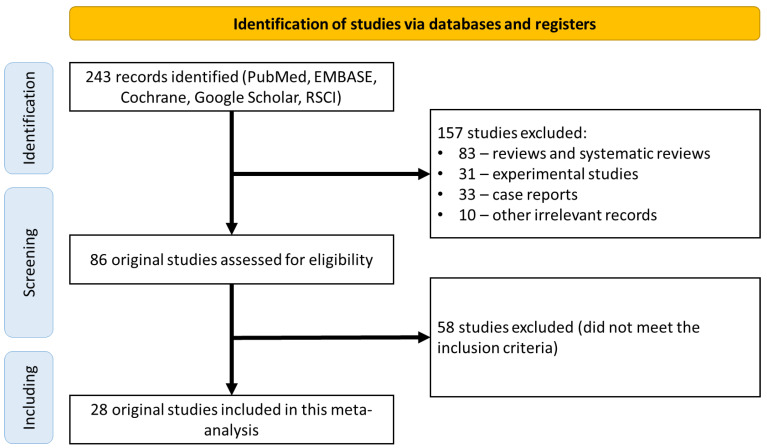
CONSORT diagram detailing the study selection strategy.

A search of the electronic databases returned 243 scientific papers for further analysis. Of these, 157 studies were excluded because they were not original clinical studies (83 reviews and systematic reviews; 31 experimental studies; 33 clinical observations; 10 other irrelevant studies). The 86 remaining studies were analyzed in detail for compliance with the inclusion criteria, which led to the exclusion of 58 studies (Figure 1). Finally, the remaining 28 original studies were considered eligible and included in the final meta-analysis (Table 2) [21,22,23,24,25,26,27,28,29,30,31,32,33,34,35,36,37,38,39,40,41,42,43,44,45,46,47,48].

### 3.2. Description of the Studies

The final analysis included 28 studies involving 4379 people (2309 patients with GERD and 2070 healthy subjects) performed in Brazil (*n* = 2) [24,39], the UK (*n* = 1) [27], Denmark (*n* = 1) [25], India (*n* = 5) [37,38,43,46,47], Iran (*n* = 1) [35], Spain (*n* = 2) [21,29], Italy (*n* = 1) [30], China (*n* = 2) [40,42], Mexico (*n* = 1) [36], Nigeria (*n* = 1) [26], Pakistan (*n* = 1) [44], Russia (*n* = 3) [28,41,48], Romania (*n* = 1) [47], Serbia (*n* = 1) [31], USA (*n* = 1) [33], Finland (*n* = 2) [22,23], France (*n* = 1) [47], and Japan (*n* = 1) [32]. The control population was represented in 21 studies [21,24,25,26,27,29,30,31,32,33,34,35,36,38,39,40,41,42,43,45,47]. In most studies, validated instrumental examination methods were used to diagnose GERD (*n* = 17) [21,22,23,24,25,26,27,28,30,32,34,35,36,38,42,43,44], and dental examination was used to diagnose DE using validated Eccles and Jenkins criteria (*n* = 7) [21,22,23,24,28,29,31], Smith and Knight (*n* = 6) [26,27,30,32,39,42], Basic Erosive Wear Examination (BEWE) (*n* = 5) [34,40,43,46,47], and the Lussi index (*n* = 2) [25,45]. The NOS assessment identified eight studies with a low risk of bias (scores of 7 or more) [21,25,27,30,39,40,42,43].

### 3.3. Prevalence of DE in GERD Patients

**Figure 2:**
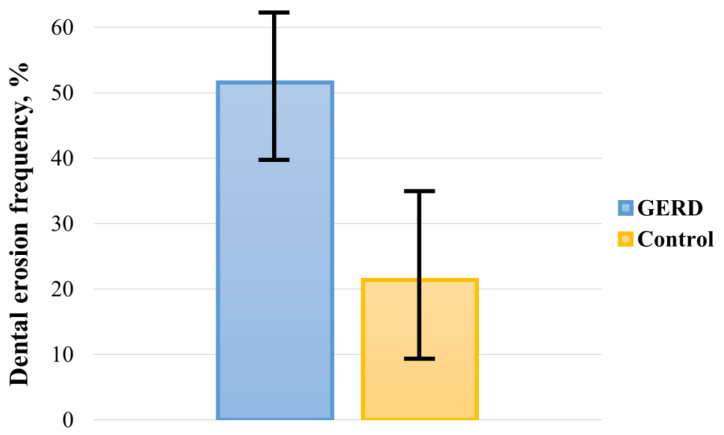
Pooled frequency of DE in patients with GERD and controls.

**Figure 3:**
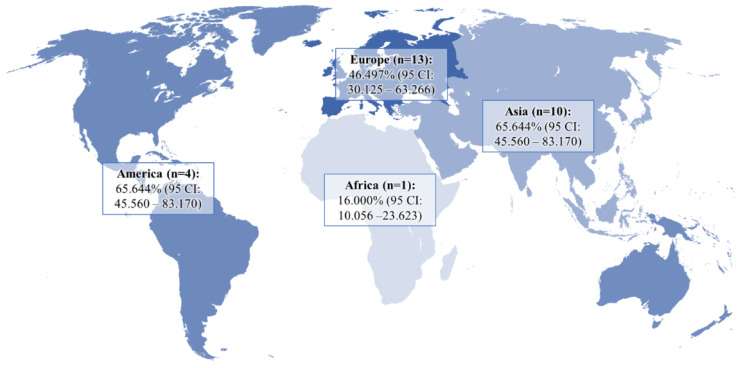
Pooled frequency of DE in patients with GERD in different regions of the world.

The pooled prevalence of DE in GERD patients and controls was 51.524% (95 CI: 39.742–63.221) and 21.351% (95 CI: 9.234–36.807), respectively (Figure 2). In the analysis, a random-effects model was used, as there was significant heterogeneity between both groups (I^2^_GERD_ = 96.95%, I^2^_control_ = 98.21%; *p* < 0.0001). Sub-analysis of the data showed that the pooled prevalence of DE in GERD patients was 46.497% (95 CI: 30.125–63.266) in Europe, 65.644% (95 CI: 45.560–83.170) in Asia, and 41.902% (95 CI: 11.019–76.927) in America (Figure 3).

### 3.4. Risk of DE in GERD Patients

**Figure 4:**
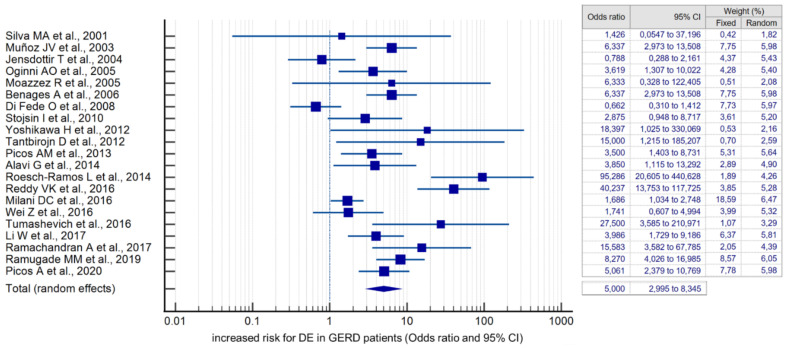
Forest plot showing the cumulative risk (OR) of DE in GERD patients [21,22,23,24,25,26,27,28,29,30,31,32,33,34,35,36,37,38,39,40,41,42,43,44,45,46,47,48].

**Figure 5:**
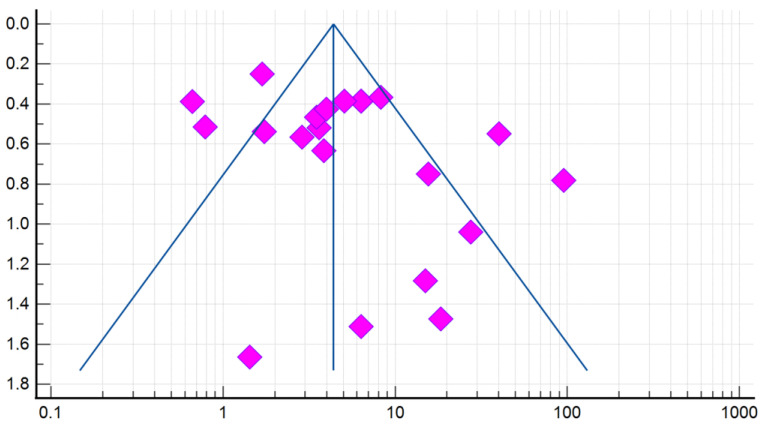
A funnel plot estimating the likelihood of a publication bias when calculating the risk (OR) of DE in patients with GERD.

Compared with controls, there was a significant association between the presence of DE and GERD according to the fixed effects model (OR 4.384, 95% CI: 3.607–5.329). However, given the high heterogeneity of the results of the included studies (I^2^ = 79.78%, 95% CI: 69.82–86.46), the risk was recalculated using a random-effects model (OR 5.000, 95% CI: 2.995–8.345) (Figure 4). When analyzing studies that used only validated instrumental methods for diagnosing GERD, alongside validated DE criteria (studies that did not specify methodologies were excluded), a significant association between the presence of DE and GERD was also revealed (OR 5.586, 95% CI: 2.311–13.503; I^2^ = 85.14%). The probability of publication bias was assessed by constructing a funnel plot and based on calculations of the Begg–Mazumdar test and the Egger’s test. A visual analysis of the funnel-shaped scattering diagram (Figure 5) did not reveal any significant asymmetry. In addition, the results of the Begg–Mazumdar test (*p* > 0.05) and the Egger’s test (*p* > 0.05) allowed for the presence of significant publication bias to be excluded.

## 4. Discussion

GERD is a widespread acid-dependent disease that develops when the motor function of the upper gastrointestinal tract is impaired [1]. Approximately one-third of patients with GERD present with atypical extraesophageal symptoms [6,7]. DE is the most common dental manifestation of GERD and is caused by persistent retrograde reflux of acidic gastric contents into the oral cavity [16,17,49]. These pathological changes in the hard tissues of the teeth are more often localized on the vestibular (buccal), occlusal, and lingual surfaces of the teeth [6].

**Figure 6:**
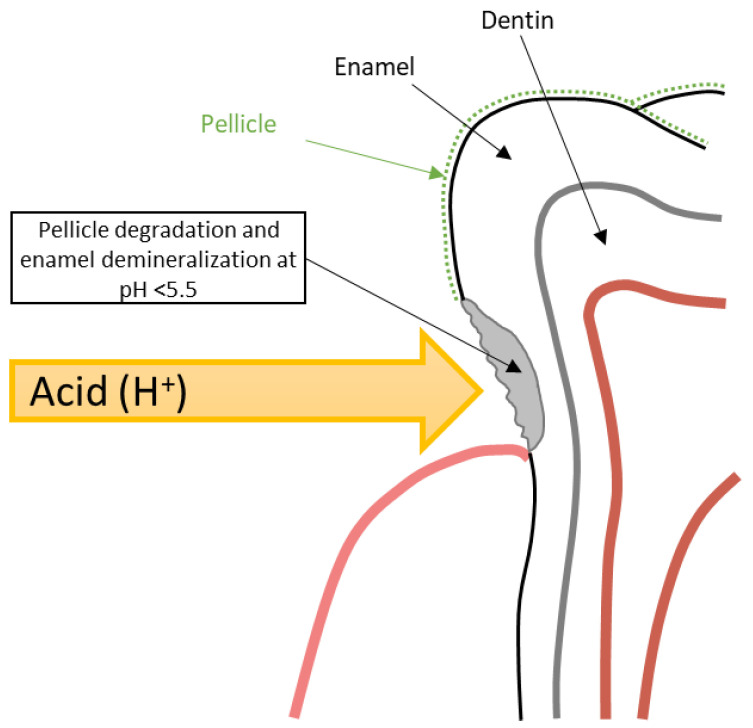
Schematic model of DE formation in GERD patients.

The development of DE within GERD occurs stage by stage. Initially, under the influence of repeated acid attacks, there is a gradual degradation of the tooth pellicle, which serves to protect the tooth hard tissue from the effects of acids [5,49]. The loss of the pellicle leads to direct contact of hydrochloric acid refluxate with the enamel surface and initiation of its demineralization at pH < 5.5 due to the dissolution of hydroxyapatite crystals (Figure 6) [49,50]. Deep DE leads to the opening of dentinal tubules and the development of hypersensitivity [5]. Saliva, which contains bicarbonates, antimicrobial substances, calcium, and phosphates, is the main protective element that can halt demineralization and promote the mineralization of dental hard tissues [50,51]. However, in GERD patients, hyposalivation is often observed, especially in obese individuals, which is also important in DE genesis [32,51].

In the studies conducted to date, the frequency of DE in GERD patients varies widely from 3.226% to 95.604% [21,22,23,24,25,26,27,28,29,30,31,32,33,34,35,36,37,38,39,40,41,42,43,44,45,46,47,48]. Through the pooling of the results of the 28 selected studies in the present meta-analysis, the pooled incidence of DE in GERD patients was determined as 51.524% (95 CI: 39.742–63.221). Moreover, compared with healthy subjects, GERD significantly increases the risk of developing DE with an OR of 5.000 (95% CI: 2.995–8.345). The data obtained are consistent with the latest systematic reviews indicating that GERD is a significant risk factor for DE [18,20,52]. In consideration of this fact, lifestyle and diet changes can be recommended for GERD patients to prevent DE (sleeping with the head of the bed raised; exclusion of excessive consumption of carbonated drinks, drinks with a low pH, sour fruits, and certain drugs). In addition to the implementation of careful individual oral hygiene (the use of rinsing agents with neutral pH), remineralizing therapy at home with the use of remineralizing gels, and regular examinations by a dentist [6,53]. Oral care products can help prevent (or at least reduce DE). There is good evidence that hydroxyapatite-containing (calcium phosphate) products are working well [54,55].

In the case of hyposalivation, it is advisable to use saliva substitutes in addition to stimulating natural salivation through the consumption of sugar-free chewing gum and specialized lozenges containing xylitol [53]. As part of DE prevention, periodic use of antacids and alginates after reflux episodes is possible. According to the latest recommendations, antisecretory therapy using proton pump inhibitors (PPIs) is the first-line therapy for the induction and maintenance of clinical remission of GERD [56,57]. With the dental manifestation of GERD, empirical observations indicate it is reasonable to use PPI therapy twice a day for three months to prevent further damage [5,6]. In a randomized controlled trial using optical coherence tomography in GERD patients with associated DE, it was shown that PPI therapy (esomeprazole 20 mg twice a day) reduces the demineralization of dental hard tissue compared with a placebo [58]. In another longitudinal non-comparative study with a follow-up period of 1 year, the use of PPIs helped in halting the progression of DE in 74% of GERD patients [59].

There are several limitations of our study. First, the studies included in the meta-analysis are characterized by significant heterogeneity in both the methods used to diagnose GERD and the criteria for diagnosing DE. Secondly, in certain studies, subjective diagnostic tools were used to diagnose GERD, e.g., questionnaires, rather than objective instrumental diagnostic methods. In addition, the limitation of this study is that the protocol of systematic review was not registered in the PROSPERO registry. However, in terms of the number of studies assessed, this meta-analysis is by far the largest to evaluate the prevalence and risk of DE in adult patients with GERD by summarizing relevant results.

## 5. Conclusions

Present meta-analysis demonstrates that DE is quite often associated with GERD and observed in about half of patients with this extremely common disease of the upper gastrointestinal tract. Given this association, it is advisable to more actively identify patients at a high risk of DE among patients with GERD and refer them to a dentist for the timely prevention and correction of this dental pathological process.

## Figures and Tables

**Table 1:** Factors leading to the development of DE.

External	Internal
Dietary factors: carbonated drinks; drinks with low pH (less than 3.5–4); fruit juices; sour fruits; ketchup and vinegar; wine; vitamin C chewable tablets/wafers.	Chronic reflux of gastric contents into the oral cavity: GERD.
2. Medications: acetylsalicylic acid; preparations of iron.	2. Recurrent vomiting: bulimia; chronic alcoholism; vomiting during pregnancy.
3. Chlorinated pool water	
4. Industrial and environmental respirable agents	

**Table 2:** Characteristics of selected studies.

Study, Year	Country	Methodology for Diagnosing GERD	Criteria for Diagnosing DE	Total GERD Patients	Total Control Persons
Järvinen, V. et al., 1988 [22]	Finland	Endoscopy	Dental examination (Eccles and Jenkins criteria)	20	NA
Meurman, J.H., et al., 1994 [23]	Finland	Endoscopy	Dental examination (Eccles and Jenkins criteria)	117	NA
Silva, M.A., et al., 2001 [24]	Brazil	Endoscopy	Dental examination (Eccles and Jenkins criteria)	31	14
Muñoz, J.V., et al., 2003 [21]	Spain	Clinical presentation + endoscopy + pH-metry	Dental examination (Eccles and Jenkins criteria)	181	72
Jensdottir, T., et al., 2004 [25]	Denmark	Clinical presentation + endoscopy + pH-metry	Dental examination (Lussi index)	23	57
Oginni, A.O., et al., 2005 [26]	Nigeria	Clinical presentation + endoscopy	Dental examination (Smith and Knight criteria)	125	100
Moazzez, R., et al., 2005 [27]	UK	Clinical presentation + pH-metry + manometry	Dental examination (Smith and Knight criteria)	31	7
Maev, I.V., et al., 2005 [28]	Russian Federation	Clinical presentation + endoscopy + pH-metry	Dental examination (Eccles and Jenkins criteria)	88	NA
Benages, A., et al., 2006 [29]	Spain	Not specified	Dental examination (Eccles and Jenkins criteria)	181	72
Di Fede, O., et al., 2008 [30]	Italy	Clinical presentation + endoscopy + pH-metry	Dental examination (Smith and Knight criteria)	200	100
Stojsin, I., et al., 2010 [31]	Serbia	Clinical presentation	Dental examination (Eccles and Jenkins criteria)	30	30
Yoshikawa, H., et al., 2012 [32]	Japan	Clinical presentation + endoscopy	Dental examination (Smith and Knight criteria)	40	30
Tantbirojn, D., et al., 2012 [33]	USA	Not specified	Optical scan	12	6
Picos, A.M., et al., 2013 [34]	Romanian	Clinical presentation + endoscopy + pH-metry	Dental examination (BEWE scale)	60	60
Alavi, G., et al., 2014 [35]	Iran	Clinical presentation + endoscopy	Dental examination	31	71
Roesch-Ramos, L., et al., 2014 [36]	Mexico	Clinical presentation + endoscopy + pH-metry + manometry	Dental examination (Eccles and Jenkins criteria)	60	60
Vinesh, E., et al., 2016 [37]	India	Not specified	Dental examination	142	NA
Reddy, V.K., et al., 2016 [38]	India	Clinical presentation + endoscopy + pH-metry	Dental examination (O’Sullivan index)	91	114
Milani, D.C., et al., 2016 [39]	Brazil	Questionnaire Symptom’s questionnaire for gastroesophageal reflux disease	Dental examination (Smith and Knight criteria)	143	274
Wei, Z., et al., 2016 [40]	China	Not specified	Dental examination (BEWE scale)	39	681
Tumashevich, O.O., et al. 2016 [41]	Russian Federation	Not specified	Dental examination	103	25
Li, W., et al., 2017 [42]	China	Clinical presentation + endoscopy	Dental examination (Smith and Knight criteria)	51	50
Ramachandran, A., et al., 2017 [43]	India	Clinical presentation + endoscopy	Dental examination (BEWE scale)	25	25
Warsi, I., et al., 2019 [44]	Pakistan	Clinical presentation + endoscopy	Dental examination	187	NA
Ramugade, M.M., et al., 2019 [45]	India	Clinical presentation	Dental examination (Lussi index)	100	100
Jacob, S., et al., 2019 [46]	India	Not specified	Dental examination (BEWE scale)	12	NA
Picos, A., et al., 2020 [47]	France, Romania	Modified GerdQ questionnaire	Dental examination (BEWE scale)	141	122
Smirnova, T.A., et al., 2021 [48]	Russian Federation	GerdQ questionnaire	Dental examination	45	NA

## Data Availability

MedCalc Database for statistical analysis: https://cloud.mail.ru/public/Y3aV/WRppBDEgM (accessed on 27 June 2022) and PRISMA statement: https://cloud.mail.ru/public/5AZG/bKGRzL4f9 (accessed on 27 June 2022).
